# Transcatheter Aortic Valve Implantation in Sievers Type 0 vs. Type 1 Bicuspid Aortic Valve Morphology: Systematic Review and Meta-Analysis

**DOI:** 10.3389/fcvm.2021.771789

**Published:** 2021-11-05

**Authors:** Yu Du, Zhijian Wang, Wei Liu, Yonghe Guo, Wei Han, Hua Shen, Shuo Jia, Yi Yu, Kangning Han, Dongmei Shi, Yingxin Zhao, Yujie Zhou

**Affiliations:** Beijing Key Laboratory of Precision Medicine of Coronary Atherosclerotic Disease, Department of Cardiology, Clinical Center for Coronary Heart Disease, Beijing Institute of Heart Lung and Blood Vessel Disease, Beijing Anzhen Hospital, Capital Medical University, Beijing, China

**Keywords:** transcatheter aortic valve implantation, Sievers type 0, outcomes, meta-analysis, bicuspid aortic valve

## Abstract

**Background:** Transcatheter aortic valve implantation (TAVI) has achieved satisfactory outcomes in the selected patients with bicuspid aortic valve (BAV), predominately type 1 BAV (~90%). However, there are few reports about the safety and efficacy of TAVI in type 0 BAV. Therefore, in the current study, we aimed to compare procedural and 30-day outcomes after TAVI between type 0 and type 1 BAV.

**Methods:** Studies comparing the outcomes of TAVI in Sievers type 0 vs. type 1 BAV were retrieved from PubMed, EMBASE, Cochrane Library, and Web of Science from inception to May 2021. The data were extracted regarding the study characteristics and outcomes. The odds ratios (ORs) with 95% CIs were pooled for procedural and 30-day outcomes.

**Results:** Six observational studies were included with determined type 0 BAV in 226 patients and type 1 BAV in 902 patients. The patients with type 0 BAV were slightly younger, had larger supra-annular structure, and more frequently implanted self-expanding prosthesis compared with type 1 BAV. In the pooled analyses, the patients with type 0 BAV had a similar incidence of procedural death (OR = 2.6, 95% CI 0.7–10.3), device success (OR = 0.6; 95% CI 0.3–1.3), and ≥ mild (OR = 0.8; 95% CI 0.4–1.6) or moderate (OR = 0.9, 95% CI 0.4–1.8) paravalvular leak, whereas significantly higher mean aortic gradient (mean difference = 1.4 mmHg, 95% CI 0.03–2.7) and increased coronary compromise risk (OR = 7.2; 95% CI 1.5–34.9), compared with type 1 BAV. Meanwhile, the incidence of death (OR = 1.2; 95% CI 0.5–3.1), stroke (OR = 0.5; 95% CI 0.1–2.4), and new pacemaker (OR = 0.6; 95% CI 0.2–2.2) at 30 days were not significantly different between the BAV morphologies (*p* > 0.05). The treatment effect heterogeneity across the studies for the above outcomes were low.

**Conclusions:** The patients with type 0 BAV appear to have similar short-term outcomes after TAVI compared with type 1 BAV. Whereas, TAVI for type 0 BAV aortic stenosis might lead to an elevated coronary obstruction risk and suboptimal aortic valvular hemodynamics.

## Introduction

Transfemoral transcatheter aortic valve implantation (TAVI) is confirmed as a safe and effective alternative to surgical aortic valve replacement (SAVR) for symptomatic, elderly patients with severe aortic stenosis (AS), regardless of the estimated surgical risk (1). However, for selected severe patients with AS and bicuspid aortic valve (BAV), TAVI has only presented a class 2b guidelines recommendation since these patients were excluded from the previous randomized controlled trials (1). Different reasons to exclude the patients with BAV in the prior trials include young age, low surgical risk, and the challenging aortic valvular complex anatomies (e.g., fused calcified raphe, asymmetric leaflet calcification, and coexisting aortopathy) (2). Recently, due to the newest generation devices and refined techniques, TAVI in the selected patients with BAV has become more prevalent, and achieved optimal procedural and short-term outcomes, except for a small, but notable, stroke and paravalvular leak (PVL) risk compared with the tricuspid aortic valves (3, 4). Meanwhile, the US Food and Drug Administration has removed the precaution from commercial labeling regarding TAVI in the patients with BAV using SAPIEN-3 (Edwards Lifesciences Inc., CA, USA) or Evolut-R/Pro (Medtronic Inc., Dublin Ireland) (5, 6).

However, BAV can present different morphologies. According to the Sievers classification, the BAV phenotypes are categorized by the raphe number (0, 1, and 2), with BAV type 1 as the most common (2, 7). The Sievers type 0 BAV morphology, with the two commissures opening in a significant elliptical fashion, was under-represented (~10%) in the previous multicenter analyses (8–10). Thus, the questions regarding the procedural and mid-term outcomes of TAVI in type 0 BAV remain unanswered. Therefore, in the present systematic review and meta-analysis, we aimed to investigate whether BAV morphology (e.g., Sievers type 0 vs. type 1) can affect the TAVI results.

## Methods

This study was performed following the Meta-Analyses of Observational Studies in Epidemiology (MOOSE) protocol (11) and reported according to the Preferred Reporting Items for Systematic Reviews and Meta-Analyses (PRISMA) checklist (12).

### Search Strategy, Study Selection, and Eligibility Criteria

According to the Population, Interventions, Comparison, Outcome and Study Design (PICOS) strategy, the studies were enrolled if the following criteria were met: (1) the population consisted of the patients with BAV that underwent TAVI; (2) there was an exposure (or intervention) group with Sievers type 0 BAV; (3) there was an exposure (or comparator) group with Sievers type 1 BAV; (4) the outcomes of interest included in-hospital, 30-day and 1-year outcomes; and (5) the comprehended observational studies. We searched for the published studies in PubMed/MEDLINE, EMBASE, Cochrane Library, and Web of Science from inception to May 2021. We used the Medical Subject Headings terms and free text to describe the following keywords: (1) “Transcatheter Aortic Valve Implantation” or “Transcatheter Aortic Valve Replacement,” (2) “Bicuspid Aortic Valve” or “Bicuspid Aortic Valve Disease,” (3) “Aortic Valve Stenosis” or “Aortic Stenosis,” and (4) “Bicuspid Aortic Valve Stenosis” or “Bicuspid Aortic Stenosis.” The search strings included: (1) AND (2), (1) AND (2) AND (3), and (1) AND (4). Some studies could have used different BAV morphological classification systems [e.g., (13, 14)] and we only included those in which the BAV classification could be translated to Sievers and Schmidtke (7). We excluded the case reports, animal studies, or studies published in non-English languages. The eligibility and quality of each study were assessed by the two independent investigators, and the discrepancies were solved by consensus.

### Data Extraction, Outcomes, and Bias Risk Assessment

We collected the following data from each study: study design, the patient characteristics, the imaging findings, the procedural details, and in-hospital, 30-day, and 1-year outcomes. The primary outcome of this study was 30-day mortality. The secondary outcomes consisted of other 30-day outcomes [stroke, life-threatening bleeding, major vascular complication, acute kidney injury (AKI) stage 2–3, and new permanent pacemaker (PPM)]; 1-year outcomes (mortality, cardiac mortality, and stroke); and in-hospital outcomes [procedural death, need of > 1 transcatheter heart valve (THV), cardiac tamponade, aortic root injury, coronary compromise, conversion to surgery, post-dilatation, new PPM, device success, and ≥ mild or moderate PVL at discharge echocardiography]. The outcomes were defined in line with the Valve Academic Research Consortium 2 (VARC-2) criteria (15). It is worth mentioning that the outcomes data were extracted only from the patients with an established Sievers type 0 or type 1 BAV anatomy. The bias risk of each study was systematically assessed using the Newcastle-Ottawa scale criteria (16).

### The Statistical Analyses

Effect summary measures are presented as the mean differences (MDs) or odds ratios (ORs) with their 95% CIs. We combined the summary measures using the random-effects Mantel–Haenszel method (17). The χ^2^ and *I*^2^ tests were used to assess the heterogeneity, with a *p* < 0.1 indicating statistical significance for heterogeneity and *I*^2^ > 50% for important heterogeneity (18). The subgroup analyses were performed in studies (1) reporting cardiac death or disabling stroke, (2) using early-generation THV (e.g., SAPIEN, SAPIEN XT, and CoreValve) vs. new-generation THV (e.g., SAPIEN-3, Evolut-R, and Evolut-Pro), and (3) using self-expanding valve (SEV) + balloon-expandable valve (BEV) vs. SEV. A sensitivity analysis was performed by removing each study from the pooled analysis in turn and examining if there was a change in the pooled results. A two-tailed *p* < 0.05 was considered statistically significant. The statistical analyses were performed using the Review Manager software (version 5.3. Cochrane Collaboration; Copenhagen, Denmark).

## Results

**Figure 1:**
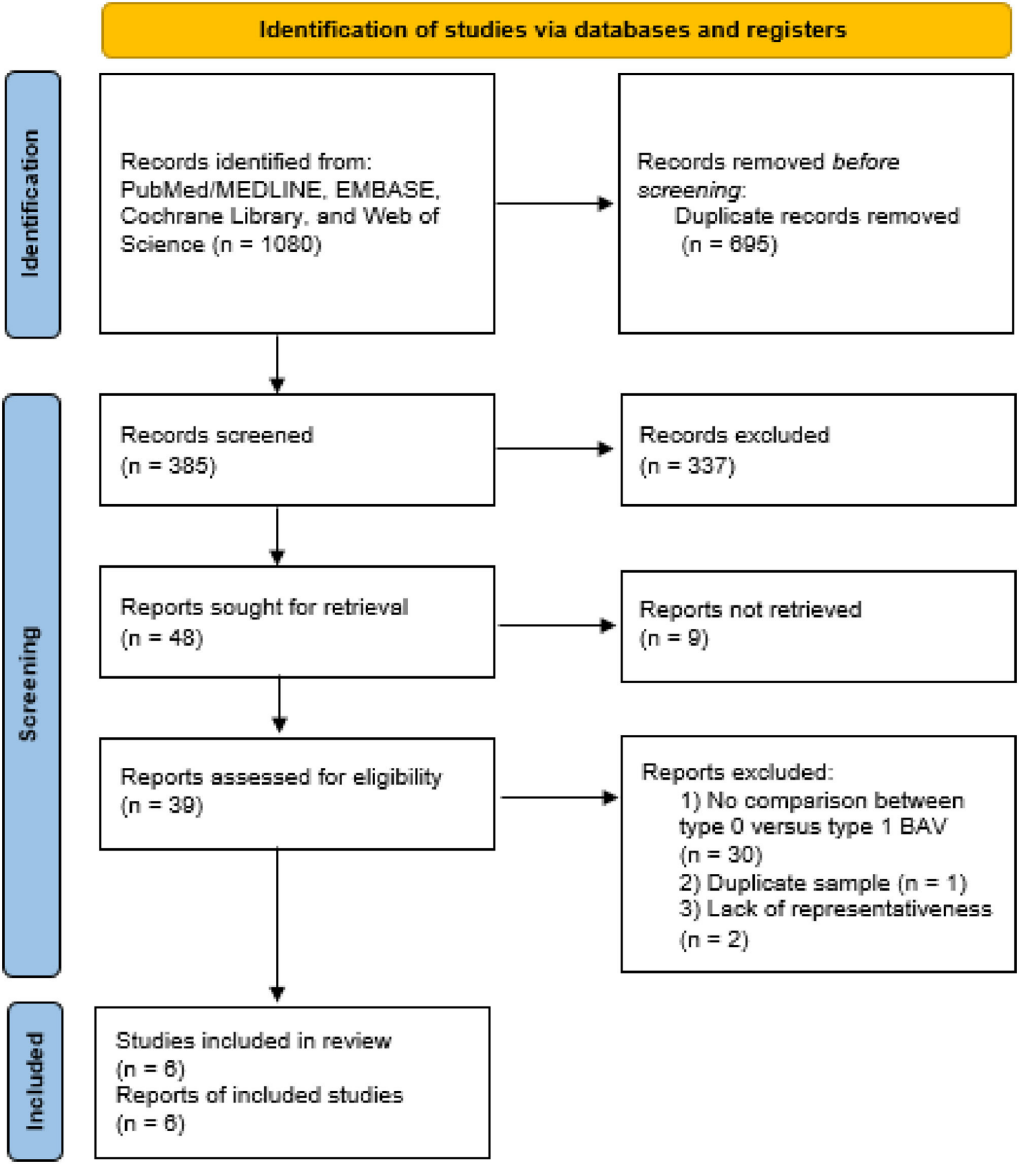
Study inclusion flowchart. BAV, bicuspid aortic valve.

The inclusion flow chart of the current study is shown in Figure 1. Six studies (1,239 patients) were enrolled to compare the procedural and clinical outcomes of TAVI between the Sievers type 0 and type 1 BAV (8, 13, 19–22). The bias risk of the enrolled studies was generally low based on the Newcastle-Ottawa scale criteria (Table 1). Multi-detector CT (MDCT) was used for BAV diagnosis in most of the patients, with 226 patients with determined Sievers type 0 BAV and 902 patients with type 1 BAV (Table 2).

**Table 1:** The risk of bias of each study by the Newcastle-Ottawa scale criteria.

	**Jilaihawi et al. (13)**	**Yoon et al. (8)**	**Liao et al. (19)**	**Fan et al. (20)**	**Forrest et al. (23)**	**Ielasi et al. (22)**
**Selection**						
-Representativeness (1)	1	1	1	1	0	1
-Non-exposed cohort (1)	1	1	1	1	1	1
-Exposure (1)	1	1	1	1	1	1
-Outcome (1)	1	1	1	1	1	1
**Comparability**						
-Most important factor (1)	0	0	0	0	0	0
-Additional factor (1)	0	0	0	0	0	0
**Outcome**						
-Assessment (1)	1	0	0	1	1	0
-Follow-up (1)	1	1	1	1	1	1
-% Follow-up (1)	1	1	1	1	1	1
Overall	7	6	6	7	6	6

**Table 2:** Overview of included BAV studies.

	**Jilaihawi et al. (13)**	**Yoon et al. (8)**	**Liao et al. (19)**	**Fan et al. (20)**	**Forrest et al. (23)**	**Ielasi et al. (22)**
Inclusion period	Apr 2005—Oct 2014	Apr 2005—May 2016	Apr 2012—Feb 2017	Dec 1—Dec 31, 2016	Dec 2018—Oct 2019	Jun 2013—Oct 2018
Location	14 centers from US, Canada, Europe and Asia	33 centers from Europe, North America and the Asia-Pacific region	1 center from China	1 center from China	25 centers from US	18 centers from Europe
Main exclusion criteria	NA	Missing data, degenerated bioprosthesis	THV neither CoreValve nor Venus-A	Absence of baseline (e.g., contraindication) or post-procedural MRI (e.g., in-hospital death, conversion to SAVR), recent stroke or TIA	STS PROM score ≥3.0%, aortopathy, age <60 yrs, prohibitive LVOT Calcium	Type 2 BAV, undeterminable BAV type
Number of patients–no.	130	546	87	83	150	243
BAV diagnosis by MDCT–no. (%)	91 (70.0)	NA	86 (98.9)	83 (100)	150 (100)	243 (100)
BAV morphology	Tricommisural BAV (*n =* 24); Bicommissural BAV (Non-raphe, *n =* 21; Raphe, *n =* 74; Undetermined, *n =* 4); Unknown (*n =* 7)	Type 0 (*n =* 61); Type 1 (*n =* 409); Type 2 (*n =* 8); Undetermined (*n =* 68)	Type 0 (*n =* 49); Type 1 (*n =* 38)	Type 0 (*n =* 56); Type 1 (*n =* 27)	Type 0 (*n =* 14); Type 1 (*n =* 136)	Type 0 (*n =* 25); Type 1 (*n =* 218)
**Type of THV**						
–Balloon expandable	Sapien or Sapien XT (*n =* 62), Sapien 3 (*n =* 8)	Sapien XT (*n =* 155), Sapien 3 (*n =* 160)	0	Sapien XT or Sapien 3 (*n =* 3)	0	Sapien 3 (*n =* 170)
–Self-expanding	CoreValve (*n =* 60)	CoreValve (*n =* 165), Evolut R (*n =* 23)	Corevalve (*n =* 25), Venus-A (*n =* 59)	CoreValve, Venus-A, VitaFlow or TaurusOne (*n =* 80)	Evolut R (*n =* 64) or Evolut PRO (*n =* 85)	Evolut R or Evolut PRO (*n =* 73)
–Others	*n =* 0	Lotus (*n =* 43)	*n =* 0	*n =* 0	*n =* 0	*n =* 0
**Mortality (%)**						
-Procedural	1.5	1.3	NA	0	0.7	0.8
-Thirty-day	3.8	3.7	9.2	0	0.7	4.0
-One-year	NA	11.4	NA	NA	NA	9.8

*BAV, bicuspid aortic valve; LVOT, left ventricular outflow tract; MRI, magnetic resonance imaging; MDCT, multi-detector computed tomography; NA, not applicable; SAVR, surgical aortic valve replacement; STS PROM, society of thoracic surgeons predicted risk of mortality; THV, transcatheter heart valve; TIA, transient ischemic attack*.

### The Baseline and Procedural Characteristics Between Sievers Type 0 and Type 1 BAV

The clinical and imaging characteristics were available for 116 patients with the Sievers type 0 BAV and 455 with type 1 BAV (Tables 3, 4). Briefly, the mean overall age of patients was 75.7 years and 39% were female. Most of the patients had New York Heart Association (NYHA) functional class III–IV (62.5%) and low society of thoracic surgeons predicted the risk of mortality (mean score of 3.7%). The patients with type 0 BAV were slightly younger (MD = −1.4 years, *p* = 0.08) and had slightly lower ejection fraction (MD = −3.9%, *p* = 0.08) compared with type 1 BAV. Notably, the patients with type 0 BAV had markedly smaller aortic valve area (MD = −0.07 cm^2^, *p* < 0.01), larger sino-tubular junction (STJ) diameter (MD = 1.9 mm, *p* < 0.01), and height (MD = 2.4 mm, *p* < 0.01), as well as larger ascending aorta diameter at 40 mm from the annulus (MD = 1.7 mm, *p* < 0.01), compared with type 1 BAV. Meanwhile, the patients with type 0 BAV had larger left (MD = 1.6 mm, *p* < 0.01) and right (MD = 1.2 mm, *p* = 0.04) coronary take-offs compared with type 1 BAV.

**Table 3:** Clinical characteristics.

	**Jilaihawi et al**. **(****13****)**		**Yoon et al. (8)**	**Liao et al. (19)**	**Fan et al**. **(****20****)**		**Forrest et al**. **(****23****)**		**Ielasi et al**. **(****22****)**		**MD or OR**	**95% CI**	***P*-value**
**BAV morphology**	**Type 0 *n =* 21**	**Type 1^#^ *n =* 74**	**Not specified *n =* 546**	**Not specified *n =* 87**	**Type 0 *n =* 56**	**Type 1 *n =* 27**	**Type 0 *n =* 14**	**Type 1 *n =* 136**	**Type 0 *n =* 25**	**Type 1 *n =* 218**			
Age (yrs)	74.4 ± 7.3	76.1 ± 10.8	77.2 ± 8.2	73.4 ± 6.4	75.0 ± 6.8	77.7 ± 3.1	70.6 ± 4.1	70.3 ± 5.6	77.8 ± 9.3	79.1 ± 7.8	−1.4	−2.9, 0.1	0.08
Male–no. (%)	11 (52.4)	46 (62.2)	343 (62.8)	50 (57.5)	33 (58.9)	16 (59.3)	5 (35.7)	73 (53.7)	19 (76.0)	144 (66.1)	0.9	0.5, 1.5	0.65
STS PROM score (%)	4.2 ± 1.6	5.1 ± 3.6	4.6 ± 4.6	7.9 ± 4.0	5.6 ± 3.6	5.8 ± 3.8	1.4 ± 0.5	1.4 ± 0.6	3.4 ± 1.8	4.5 ± 3.0	−0.5	−1.2, 0.2	0.15
NYHA class III-IV–no. (%)	18 (85.7)	60 (81.1)	439 (80.4)	80 (92.0)	51 (91.1)	24 (88.9)	2 (14.3)	39 (28.6)	17 (68.0)	146 (67.3)	1.0	0.5, 1.8	0.98
Prior PCI–no. (%)	4 (19.0)	8 (10.8)	121 (22.2)	7 (8.0)	3 (5.4)	5 (18.5)	1 (7.1)	10 (7.4)	6 (24.0)	54 (24.8)	0.9	0.4, 1.9	0.73
Prior CABG–no. (%)	1 (4.8)	8 (10.8)	62 (11.4)	NA	0 (0)	0 (0)	2 (14.3)	0 (0)	2 (8.0)	18 (8.3)	2.1	0.2, 21.2	0.54
CKD–no. (%)	1 (5.0)^*^	19 (29.7)^*^	NA	10 (16.1)	NA	NA	NA	NA	NA	NA	NA	NA	NA
Lung disease–no. (%)	6 (28.6)	31 (41.9)	98 (17.9)	50 (57.5)	13 (23.2)	6 (22.2)	2 (15.4)	24 (17.9)	7 (28)	52 (23.9)	0.9	0.5, 1.6	0.72
Stroke or TIA–no. (%)	3 (14.3)	9 (12.2)	77 (14.1)	13 (14.9)	0 (0)	1 (3.7)	0 (0)	10 (7.4)	4 (16.0)	27 (12.4)	1.0	0.5, 2.3	0.95
Atrial fibrillation or flutter—no. (%)	6 (28.6)	24 (32.4)	NA	19 (21.8)	5 (18.5)	11 (13.3)	0 (0)	11 (8.1)	6 (25.0)	54 (25.5)	0.5	0.2, 1.3	0.16
Prior PPM—no. (%)	2 (9.5)	12 (16.2)	NA	NA	NA	NA	0 (0)	4 (2.9)	2 (8.0)	20 (9.2)	0.9	0.2, 3.4	0.87

*Values are mean ± SD, median (interquartile range) or n (%)*.

# *Functional (or tricommisural) BAV not included;*

* *indicated statistically significant difference (P < 0.05) between type 0 and type 1 within the study*.

*BAV, bicuspid aortic valve; CI, Confidence Interval; CABG, coronary artery bypass graft; CKD, chronic kidney disease; MD, Mean Difference; NYHA, New York Heart Association; NA, not applicable; PCI, percutaneous coronary intervention; PPM, permanent pacemaker; STS PROM, society of thoracic surgeons predicted risk of mortality; TIA, transient ischemic attack*.

**Table 4:** Imaging findings.

	**Jilaihawi et al**. **(****13****)**		**Yoon et al. (8)**	**Liao et al. (19)**	**Fan et al**. **(****20****)**		**Forrest et al**. **(****23****)**		**Ielasi et al**. **(****22****)**		**MD or OR**	**95% CI**	***P*-value**
**BAV morphology**	**Type 0 *n =* 21**	**Type 1^#^ *n =* 74**	**Not specified *n =* 546**	**Not specified *n =* 87**	**Type 0 *n =* 56**	**Type 1 *n =* 27**	**Type 0 *n =* 14**	**Type 1 *n =* 136**	**Type 0 *n =* 25**	**Type 1 *n =* 218**			
**Echocardiography**													
Mean aortic gradient (mmHg)	50.3 ± 14.3	50.8 ± 15.9	49.7 ± 17.7	65.4 ± 20.1	56.3 ± 25.7	51.7 ± 12.5	48.1 ± 9.7	50.0 ± 16.0	46.0 ± 10.4	49.2 ± 16.8	−1.3	−4.3, 1.7	0.39
AVA (cm^2^)	0.60 ± 0.24	0.67 ± 0.19	0.70 ± 0.20	NA	0.50 ± 0.18	0.57 ± 0.23	0.70 ± 0.10	0.80 ± 0.20	0.67 ± 0.22	0.69 ± 0.23	−0.07	−0.12, −0.03	** <0.01**
Moderate/severe AR—no. (%)	NA	NA	NA	12 (13.8)	6 (10.7)	5 (18.5)	NA	NA	4 (16.0)	46 (21.1)	0.6	0.3, 1.5	0.28
Ejection fraction (%)	NA	NA	51.6 ± 15.0	55.0 ± 19.6	55.2 ± 15.2	58.1 ± 9.2	NA	NA	48.8 ± 15.5	54.2 ± 13.2	−3.9	−8.0, 0.1	0.06
**MDCT**													
Aortic root angle (degree)	50.1 ± 10.6	50.8 ± 11.4	NA	NA	52.8 ± 9.8	52.7 ± 8.4	NA	NA	NA	NA	−0.2	−3.4, 3.0	0.90
Calcium score (mm^3^)	546.3 ± 645.6	391.3 ± 283.5	NA	654.8 ± 406.1	995.1 ± 781.4	919.2 ± 343.4	491.5 ± 425.2	817.2 ± 563.8	NA	NA	−36.7	−332.0, 258.7	0.81
Moderate/severe aortic valve calcium—no. (%)	NA	NA	NA	NA	NA	NA	NA	NA	13 (52.0)^*^	155 (71.1)^*^	NA	NA	NA
Annulus area (mm^2^)	434.4 ± 92.7^*^	505.0 ± 93.3^*^	NA	459.3 ± 136.4	462.0 ± 118.8	442.1 ± 75.2	NA	NA	547.2 ± 133.2	509.2 ± 107.3	−5.0	−71.0, 60.9	0.88
Annular perimeter (mm)	75.0 ± 8.1^*^	80.9 ± 7.5^*^	NA	78.0 ± 9.5	77.8 ± 9.6	76.3 ± 5.9	NA	NA	83.4 ± 10.8	81.4 ± 8.9	−0.8	−5.8, 4.2	0.75
STJ diameter (mm)	33.5 ± 6.0	32.0 ± 4.2	NA	30.8 ± 3.9	31.8 ± 3.7	28.7 ± 4.1	NA	NA	31.0 ± 3.6	30.0 ± 4.3	1.9	0.5, 3.2	** <0.01**
STJ height (mm)	26.4 ± 5.1	24.4 ± 4.8	NA	NA	24.5 ± 5.4	21.8 ± 5.3	NA	NA	NA	NA	2.4	0.6, 4.1	** <0.01**
AAo diameter at 4 cm (mm)	38.8 ± 5.8	37.7 ± 5.0	NA	NA	38.9 ± 3.4	37.1 ± 2.3	NA	NA	NA	NA	1.7	0.6, 2.8	** <0.01**
Max AAo diameter (mm)	42.5 ± 6.4	40.5 ± 6.5	NA	NA	43.9 ± 4.0	39.2 ± 2.8	NA	NA	36.6 ± 4.0	36.8 ± 5.4	2.2	−1.2, 5.6	0.21
Left coronary height (mm)	15.5 ± 4.3	14.5 ± 3.6	NA	14.1 ± 3.5	17.2 ± 3.9	15.3 ± 2.0	NA	NA	NA	NA	1.6	0.6, 2.7	** <0.01**
Right coronary height (mm)	17.9 ± 2.9	17.1 ± 3.7	NA	15.3 ± 3.1	17.5 ± 4.3	15.8 ± 3.5	NA	NA	NA	NA	1.2	0.1, 2.3	**0.04**

*Values are mean ± SD, median (interquartile range) or n (%)*.

# *Functional (or tricommisural) BAV not included;*

* *indicated statistically significant difference (P < 0.05) between type 0 and type 1 within the study*.

*AVA, aortic valve area; AR, aortic regurgitation; AAo, ascending aorta; BAV, bicuspid aortic valve; CABG, coronary artery bypass graft; CI, Confidence Interval; CKD, chronic kidney disease; MD, Mean Difference; NYHA, New York Heart Association; NA, not applicable; PCI, percutaneous coronary intervention; PPM, permanent pacemaker; STJ, sino-tubular junction; STS PROM, society of thoracic surgeons predicted risk of mortality; TIA, transient ischemic attack*.

*Bold values indicated statistically significant difference (P < 0.05) between type 0 and type 1 in pooled analysis*.

The TAVI procedural details were available for 156 patients with Sievers type 0 BAV and 790 with type 1 BAV (Table 5). Overall, the conscious sedation (68.9%) and balloon pre-dilation (73.0%) were commonly used. Most of the patients (90.0%) had transfemoral access and nearly half (50.8%) implanted SEV. It is worth noting that, compared with type 1 BAV, the patients with type 0 BAV were less likely to implant BEV (OR = 0.5, 95% CI 0.2–0.9), and numerically more frequently received SEV (OR = 2.2, 95% CI 0.9–4.8).

**Table 5:** Procedural details.

	**Jilaihawi et al. (13)**	**Yoon et al**. **(****8****)**		**Liao et al. (19)**	**Fan et al**. **(****20****)**		**Forrest et al**. **(****23****)**		**Ielasi et al**. **(****22****)**		**MD or OR**	**95% CI**	***P*-value**
**BAV morphology**	**Not specified *n =* 130**	**Type 0 *n =* 61**	**Type 1 *n =* 409**	**Not specified *n =* 87**	**Type 0 *n =* 56**	**Type 1 *n =* 27**	**Type 0 *n =* 14**	**Type 1 *n =* 136**	**Type 0 *n =* 25**	**Type 1 *n =* 218**			
Conscious sedation—no. (%)	NA	NA	NA	4 (4.6)	47 (83.9)	24 (88.9)	9 (64.3)	86 (63.2)	20 (80.0)	198 (90.8)	0.6	0.3, 1.3	0.20
Transfemoral access—no. (%)	114 (87.7)	50 (82.0)	360 (88.0)	83 (95.4)	NA	NA	14 (100)	133 (98.5)	25 (100)	193 (88.5)	1.0	0.3, 3.7	0.97
Pre-dilation—no. (%)	116 (91.3)	NA	NA	81 (93.1)	56 (100)	27 (100)	14 (100)	123 (90.4)	11 (44.0)	78 (35.8)	1.50	0.7, 3.4	0.32
**THV type—no. (%)**													
–Self-expanding	60 (46.2)	32 (52.4)^*^	113 (27.6)^*^	84 (96.5)	56 (100)	24 (88.9)	14 (100)	135 (100)	9 (36.0)	64 (29.4)	2.2	0.9, 4.8	0.06
–Balloon expandable	70 (53.8)	25 (41.0)^*^	260 (63.6)^*^	0 (0)	0 (0)	3 (11.1)	0 (0)	0 (0)	16 (64.0)	154 (70.6)	0.5	0.2, 0.9	**0.03**
–Mechanically expandable	0 (0)	4 (6.6)^*^	36 (8.8)^*^	0 (0)	0 (0)	0 (0)	0 (0)	0 (0)	0 (0)	0 (0)	NA	NA	NA

* *indicated statistically significant difference (P < 0.05) between type 0 and type 1 within the study*.

*BAV, bicuspid aortic valve; CI, Confidence Interval; MD, Mean Difference; NA, not applicable; THV, transcatheter heart valve 0.591–1,163*.

*Bold values indicated statistically significant difference (P < 0.05) between type 0 and type 1 in pooled analysis*.

### The Procedural and Clinical Outcomes Between Sievers Type 0 and Type 1 BAV

**Figure 2:**
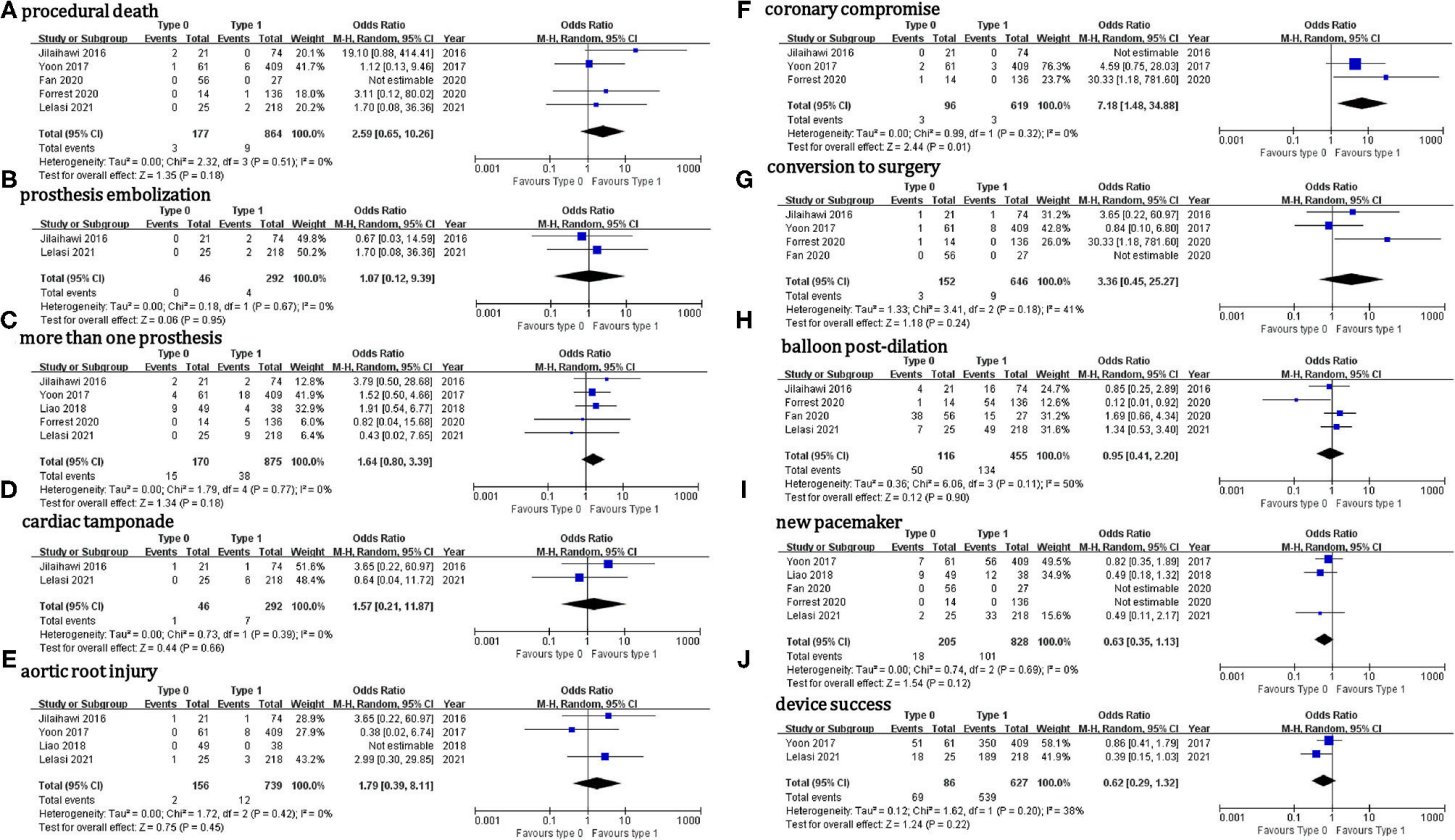
In-hospital outcomes.

Outcome data were available for 226 patients with Sievers type 0 BAV and 902 with type 1 BAV (Table 6). Regarding the in-hospital outcomes, no significant difference was observed for the patients with Sievers type 0 vs. type 1 BAV that underwent TAVI: procedural death (OR = 2.6, 95% CI 0.7–10.3), THV embolization (OR = 1.1, 95% CI 0.11–9.4), > 1 THV (OR = 1.6, 95% CI 0.8–3.4), cardiac tamponade (OR = 1.6, 95% CI 0.2–11.9), aortic root injury (OR = 1.8, 95% CI 0.4–8.1), conversion to surgery (OR = 3.4, 95% CI 0.5–25.3), balloon post-dilation (OR = 0.95, 95% CI 0.4–2.2), new PPM (OR = 0.6, 95% CI 0.4–1.1), device success (OR = 0.6, 95% CI 0.3–1.3), ≥ mild (OR = 0.8, 95% CI 0.4–1.6), or ≥ moderate PVL (OR = 0.9, 95% CI 0.4–1.8) (Figure 2). It is worth mentioning that, compared with type 1 BAV, TAVI for the patients with type 0 BAV was associated with significant higher mean aortic gradient (MD = 1.35 mmHg, 95% CI 0.03–2.7) and increased coronary compromise risk (OR = 7.2; 95% CI 1.5–34.9). The treatment effect heterogeneity was low across the studies for the above outcomes, except for balloon post-dilation among the four studies with a borderline heterogeneity (*p* = 0.11, *I*^2^ = 50%).

**Table 6:** In-hospital and 30-day outcomes.

	**Jilaihawi et al**. **(****13****)**		**Yoon et al**. **(****8****)**		**Liao et al**. **(****19****)**		**Fan et al**. **(****20****)**		**Forrest et al**. **(****23****)**		**Ielasi et al**. **(****22****)**	
**BAV morphology**	**Type 0**	**Type 1^#^**	**Type 0**	**Type 1**	**Type 0**	**Type 1**	**Type 0**	**Type 1**	**Type 0**	**Type 1**	**Type 0**	**Type 1**
	***n =* 21**	***n =* 74**	***n =* 61**	***n =* 409**	***n =* 49**	***n =* 38**	***n =* 56**	***n =* 27**	***n =* 14**	***n =* 136**	***n =* 25**	***n =* 218**
**In-hospital outcomes—no. (%)**												
Procedural death	2 (9.5)^*^	0 (0)^*^	1 (1.6)	6 (1.5)	NA	NA	0 (0)	0 (0)	0 (0)	1 (0.7)	0 (0)	2 (0.9)
Prosthesis embolization	0 (0)	2 (2.7)	NA	NA	NA	NA	NA	NA	NA	NA	0 (0)	2 (0.9)
Need of > 1 prosthesis	2 (9.5)	2 (2.7)	4 (6.6)	18 (4.4)	9 (18.4)	4 (11.8)	NA	NA	0 (0)	5 (3.7)	0 (0)	9 (4.1)
Cardiac tamponade	1 (4.8)	1 (1.4)	NA	NA	NA	NA	NA	NA	NA	NA	0 (0)	6 (2.8)
Aortic root injury	1 (4.8)	1 (1.4)	0 (0)	8 (2.0)	0 (0)	0 (0)	NA	NA	NA	NA	1 (4.0)	3 (1.4)
Coronary compromise	0 (0)	0 (0)	2 (3.3)	3 (0.7)	NA	NA	NA	NA	1 (7.1)	0 (0)	NA	NA
Conversion to surgery	1 (4.8)	1 (1.4)	1 (1.6)	8 (2.0)	NA	NA	0 (0)	0 (0)	1 (7.1)	0 (0)	NA	NA
Post-dilation	4 (19.0)	16 (22.2)	NA	NA	NA	NA	38 (67.9)	15 (55.6)	1 (7.1)	54 (40.0)	7 (28.0)	49 (22.5)
New PPM	NA	NA	7 (11.5)	56 (14.4)	9 (18.4)	12 (31.6)	0 (0)	0 (0)	0 (0)	0 (0)	2 (8.0)	33 (15.5)
Device success	NA	NA	51 (83.6)	350 (85.6)	NA	NA	NA	NA	NA	NA	18 (72.0)	189 (86.7)
**Pre-discharge echocardiography**												
≥ mild PVL—no. (%)	12 (60.0)	49 (68.1)	NA	NA	19 (38.8)	15 (41.2)	NA	NA	NA	NA	NA	NA
≥ moderate PVL—no. (%)	3 (15.0)	14 (19.4)	5 (8.2)	44 (10.8)	NA	NA	6 (10.7)	0 (0)	0 (0)	0 (0)	1 (4.0)	9 (4.1)
Mean aortic gradient (mmHg)^**§**^	10.0 (7.0–14.0)	9.5 (7.8–13.0)	12.0 ± 7.2	10.4 ± 5.1	NA	NA	NA	NA	NA	NA	11.5 ± 6.7	9.4 ± 4.7
**Thirty-day outcomes–no. (%)**												
All-cause mortality	2 (9.5)	2 (2.7)	1 (1.6)	17 (4.2)	5 (10.2)	3 (7.9)	0 (0)	0 (0)	0 (0)	1 (0.7)	0 (0)	9 (4.4)
-Cardiac mortality	NA	NA	NA	NA	NA	NA	0 (0)	0 (0)	0 (0)	1 (0.7)	0 (0)	8 (3.9)
Stroke	0 (0)	3 (4.2)	0 (0)	13 (3.2)	NA	NA	NA	NA	0 (0)	6 (4.4)	0 (0)	3 (1.5)
-Disabling	NA	NA	0 (0)	8 (2.1)	NA	NA	NA	NA	0 (0)	1 (0.7)	NA	NA
-Non-disabling	NA	NA	0 (0)	5 (1.3)	NA	NA	NA	NA	0 (0)	5 (3.7)	NA	NA
Life threatening bleeding	NA	NA	0	8 (2.0)	NA	NA	NA	NA	0 (0)	6 (4.4)	NA	NA
Major vascular complication	NA	NA	0	14 (3.4)	NA	NA	NA	NA	0 (0)	2 (1.5)	NA	NA
AKI stage 2−3	NA	NA	1 (1.6)	9 (2.2)	NA	NA	NA	NA	0 (0)	0 (0)	NA	NA
New PPM	4 (22.2)	16 (26.7)	NA	NA	NA	NA	NA	NA	0 (0)	22 (16.7)	NA	NA
**One-year outcomes–no. (%)**												
All-cause mortality	NA	NA	NA	NA	NA	NA	NA	NA	NA	NA	2 (9.1)	18 (9.8)
-Cardiac mortality	NA	NA	NA	NA	NA	NA	NA	NA	NA	NA	2 (9.1)	13 (7.1)
Stroke	NA	NA	NA	NA	NA	NA	NA	NA	NA	NA	1 (5.6)	7 (7.6)

*Values are mean ± SD, median (interquartile range) or n (%)*.

# *Functional (or tricommisural) BAV not included;*

* *indicated statistically significant difference (P < 0.05) between type 0 and type 1 within the study; §Mean difference between type 0 and type 1 BAV of 1.35 [0.03–2.66], I^2^ = 0%, P = 0.05*.

*AKI, acute kidney injury; BAV, bicuspid aortic valve; NA, not applicable; PPM, permanent pacemaker; PVL, paravalvular leak*.

**Figure 3:**
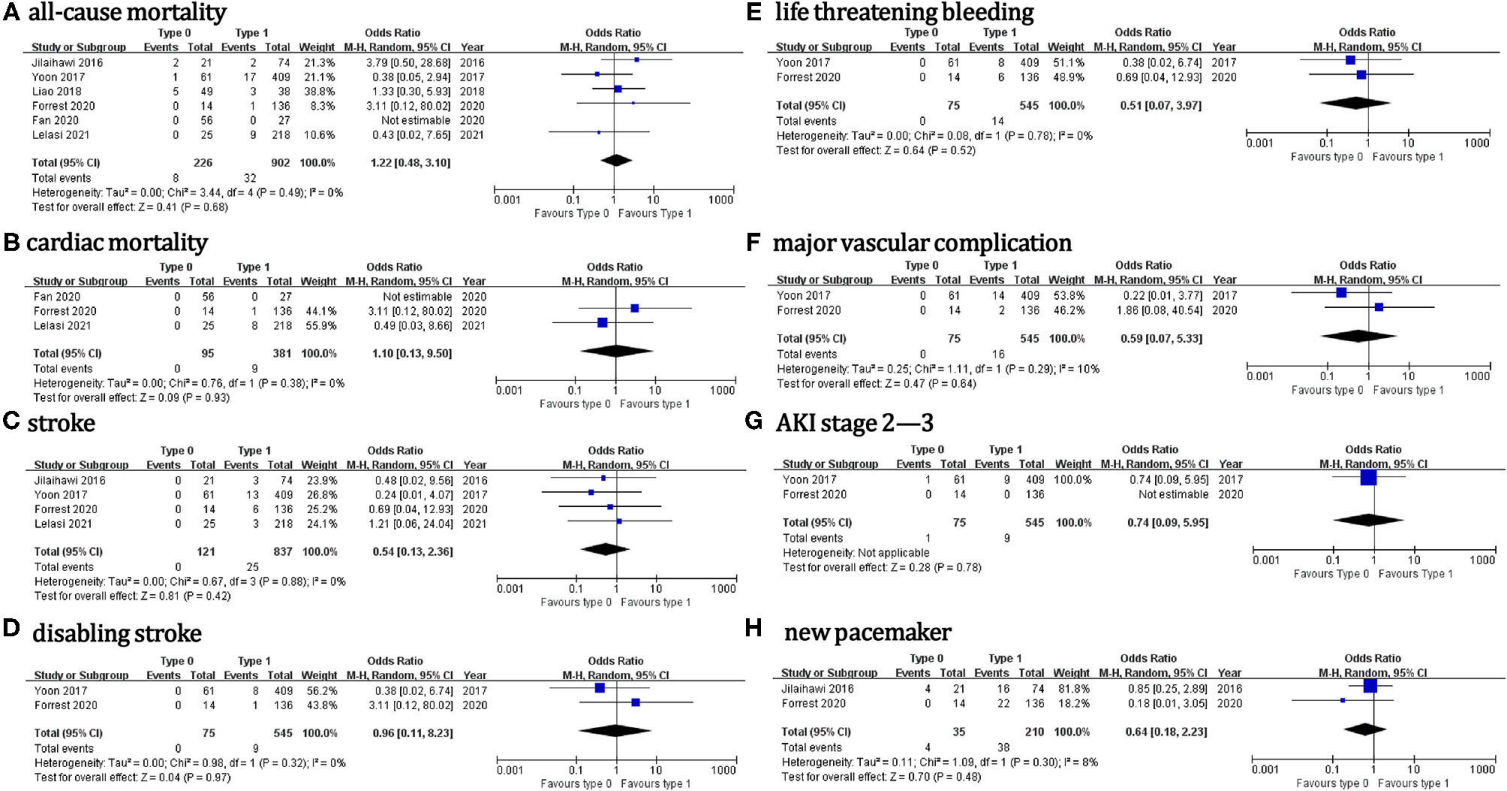
The 30-day outcomes.

Regarding the 30-day outcomes (Figure 3), we did not found significant differences in TAVI for patients with type 0 vs. type 1 BAV: all-cause death (OR = 1.2, 95% CI 0.5–3.1), cardiac death (OR = 1.1, 95% CI 0.1–9.5), stroke (OR = 0.5, 95% CI 0.1–2.4), disabling stroke (OR = 0.96, 95% CI 0.1–8.2), life threatening bleeding (OR = 0.5, 95% CI 0.1–4.0), major vascular complication (OR = 0.6, 95% CI 0.1–5.3), AKI stage 2–3 (OR = 0.7, 95% CI 0.1–6.0) or new PPM (OR = 0.6, 95% CI 0.2–2.2). No significant treatment effect heterogeneity was found among the studies for these outcomes. Additionally, the pooled results were almost unchanged in the sensitivity analysis.

One-year outcomes were available in only one study (22), showing no difference in all-cause mortality, cardiac mortality, and stroke between the two BAV phenotypes (*p* > 0.05).

### The Subgroup Analyses Between the Sievers Type 0 and Type 1 BAV

No significant differences in the procedural and 30-day outcomes between TAVI in the patients with type 0 and type 1 BAV were observed using either early-generation THV (e.g., SAPIEN, SAPIEN XT, and CoreValve) or new-generation THV (e.g., SAPIEN-3, Evolut-R, and Evolut-Pro) (Table 7), and using either SEV + BEV or SEV (Table 8).

**Table 7:** Subgroup analyses of different generations of prosthesis^*^.

	**Early-generation prosthesis**				**New-generation prosthesis**			
	**Sapien, Sapien XT, CoreValve**				**Sapien 3, Evolut R, Evolut PRO**			
	**OR**	**95% CI**	***I^**2**^***	***P-*value**	**OR**	**95% CI**	***I* ^**2**^**	***P-*value**
**30-day outcomes**
Death	1.9	0.6–6.4	0	0.29	1.0	0.1–8.9	0	0.98
Stroke	NA	NA	NA	NA	0.9	0.1–7.4	0	0.93
**In-hospital outcomes**
Procedural death	19.1	0.9–414.4	NA	0.06^§^	2.3	0.2–21.0	0	0.47
>1 prosthesis	2.3	0.8–6.8	0	0.12	0.6	0.1–4.6	0	0.62
Aortic root injury	3.7	0.2–61.0	NA	0.37^§^	NA	NA	NA	NA
Conversion to surgery	3.7	0.2–61.0	NA	0.37^§^	NA	NA	NA	NA
Post-dilation	1.3	0.6–2.8	0	0.48	0.5	0.04–5.7	80%	0.55
Pacemaker	0.5	0.2–1.3	NA	0.16^§^	0.5	0.1–2.2	NA	0.35^§^
Perivalvular leak ≥ moderate	1.6	0.2–14.5	52%	0.69	1.0	0.1–8.0	NA	0.98^§^

* *The study by Yoon et al. (8) was not included in either of the two subgroups because 58.6 and 41.4% of patients used early- and new-generation prosthesis, respectively*.

§ *Two studies were eligible for pool analysis, whereas in one study, no event occurred in type 0 or type 1 BAV*.

*CI, confidence interval; OR, odds ratio*.

**Table 8:** Subgroup analyses of different types of prosthesis.

	**SEV+BEV**				**SEV**			
	**OR**	**95% CI**	***I* ^**2**^**	***P-*value**	**OR**	**95% CI**	***I* ^**2**^**	***P-*value**
**30-day outcomes**
Death	1.0	0.2–4.7	33%	0.95	1.54	0.4–6.0	0	0.53
Stroke	0.5	0.1–2.7	0	0.43	NA	NA	NA	NA
**In–hospital outcomes**
Procedural death	2.6	0.5–13.7	14%	0.26	3.1	0.1–80.0	NA	0.49^§^
>1 prosthesis	1.6	0.6–4.1	0	0.31	1.7	0.5–5.4	0	0.38
Aortic root injury	1.8	0.4–8.1	0	0.45	NA	NA	NA	NA
Coronary compromise	4.6	0.8–28.0	NA	0.10^§^	NA	NA	NA	NA
Conversion to surgery	1.4	0.3–7.6	0	0.69	30.3	1.2–781.6	NA	0.04^§^
Post-dilation	1.1	0.5–2.4	0	0.73	0.5	0.03–8.0	83%	0.63
Pacemaker	0.7	0.4–1.5	0	0.38	0.5	0.2–1.3	NA	0.16^§^
Perivalvular leak ≥ moderate	0.8	0.4–1.6	0	0.46	7.1	0.4–130.4	NA	0.19^§^

§ *Two studies were eligible for pool analysis, whereas in one study, no event occurred in type 0 or type 1 BAV*.

*BEV, balloon expandable valve; SEV, self-expanding valve; CI, confidence interval; OR, odds ratio*.

## Discussion

To our knowledge, this study comprehended the first meta-analysis comparing the procedural and clinical outcomes of TAVI in the patients with Sievers type 0 vs. type 1 BAV. Our main findings were: (1) the incidence of most procedural outcomes was similar between the type 0 vs. type 1 BAV (i.e., procedural death, THV embolization, > 1 THV, cardiac tamponade, aortic root injury, conversion to surgery, balloon post-dilation, new PPM, device success, ≥ mild PVL, or ≥ moderate PVL). However, the patients with type 0 BAV were associated with markedly higher mean aortic gradient before discharge and increased coronary compromise risk compared with type 1 BAV. (2) No marked differences between the two BAV configurations were found for the following 30-day outcomes: death, cardiac death, stroke, disabling stroke, life-threatening bleeding, major vascular complication, AKI stage 2–3, or new PPM. Importantly, the treatment effect heterogeneity was consistently low across the studies for procedural and 30-day outcomes. (3) The subgroup analyses in the patients using different THV generations, different THV types, and different hard endpoints definitions were consistent with the aforementioned procedural and 30-day outcomes.

Bicuspid aortic valve is the most common congenital heart disease (1~2% of the population) and represents the main AS cause in the patients under 65 years of age (24, 25). Given the indications of TAVI expanding to the young patients with AS, more patients with BAV can be encounter in the contemporary pre-TAVI workup. However, little is known about the correlation between the Sievers BAV phenotypes and the clinical manifestations and outcomes after TAVI. Data from a large international, multicenter registry (*n* = 2,118) showed that, compared with BAV with raphe, the patients with BAV without raphe (i.e., type 0 BAV) referring for cardiac surgery were younger, less likely to have dysfunctional aortic valves, whereas had similar Valsalva sinus, STJ, and ascending aorta diameters by ECGs (26). In contrast, we found, in 571 patients with BAV that underwent TAVI for severe AS, that patients with type 0 BAV were only slightly younger and had numerically lower ejection fraction compared with type 1 BAV. The patients with BAV in the present study appeared much older (75.7 vs. 47.0 years) and to have more frequently severe AS (100 vs. 19.6%) than the aforementioned surgical registry (26). Meanwhile, we found a significantly larger STJ diameter and height, as well as ascending aorta diameter at 40 mm from the annulus. In addition, Jilaihawi et al. showed that the mean Valsalva sinus diameter was larger in the type 0 BAV than type 1 (13). Consistently, these findings demonstrated that type 0 BAV was associated with a larger supra-annular structure than type 1 BAV.

Regarding the TAVI procedure, the balloon pre-dilation proportion was high whereas varied among the different centers [93.1~100% in two Chinese centers (19, 20) and 36.6% in an international registry mainly compromising the European centers (22)]. Balloon valvuloplasty for BAV-AS is supposed to facilitate the TAVI delivery system crossing, improve prosthesis expansion, and judge prosthesis size selection and coronary obstruction risk in combination with aortography (2). However, routine balloon pre-dilation might increase procedural stroke (20), and the benefit of deploying a cerebral embolic protection device remains to be established in this scenario. In our present pooled analysis, the 30-day stroke risk was similar between the type 0 and type 1 BAV, although no patient had a 30-day stroke in the type 0 BAV group (8, 13, 22, 23). Consistent with these findings, Fan et al. demonstrated a similar number and total volume of cerebral ischemic lesions in diffusion-weighted MRI after TAVI between the two BAV categories (20).

Interestingly, the patients with type 0 BAV seemed more likely to implant SEV than BEV. This might be explained by the fact that TAVI for BAV-AS using BEV was associated with more than a five-time higher annulus rupture risk than SEV (2.5% vs. 0, *p* < 0.001) (27). Moreover, type 0 BAV is uncommon in clinical practice, where the physicians might not be well-experienced with this specific aortic morphology and thus tend to frequently use SEV. Although TAVI for BAV-AS using SEV, compared with BEV, was associated with a higher tolerable error rate, it might also lead to an increased moderate or severe PVL risk, probably due to the decreased radial force of SEV (28). Moderate or severe PVL is a major concern in the early trials of performing TAVI in BAV (incidence ranging from 8 to 20%) (8, 13). Fortunately, its incidence significantly decreased (<4%) due to a more precise aortic valve sizing by MDCT and the use of new-generation THV with an extra sealing skirt or re-capture property (22, 23). In our analysis, although the mean aortic gradient on pre-discharge echocardiography was markedly higher in type 0 compared with type 1 BAV, this small difference on aortic gradient (MD = 1.35 mmHg) did not lead to the significant differences in the ≥ mild or ≥ moderate PVL incidence between the two BAV groups. It is worth mentioning that the impact of prosthesis selection (BEV vs. SEV or early- vs. new-generation) on the procedural outcomes should be treated as hypothesis-generating at this time since we did not observe these impacts in our subgroup analysis.

Although the patients with type 0 BAV tended to have larger supra-annular structures and higher coronary take-offs, we found that TAVI for type 0 BAV was associated with a significantly higher coronary compromise risk compared with type 1. Traditionally, the coronary obstruction predictors during TAVI include low coronary take-off, small Valsalva sinus and STJ, long aortic leaflet, and bulky calcification close to the coronary ostium. Recently, Heitkemper et al. found that the distance ratio from cusp to coronary ostium to coronary artery diameter (<0.7) was superior to coronary ostium height (< 14 mm) and Valsalva sinus diameter (< 30 mm) to predict coronary obstruction in TAVI, with 100% sensitivity and 95.7% specificity (29). Thus, predicting coronary obstruction during TAVI can be difficult, in particular, for the challenging BAV anatomies. Meanwhile, coronary access post-TAVI is important considering that the patients with BAV-AS are generally young and at low surgical risk. In this case, BEV with an intra-annular and lower-frame design can be more friendly than SEV allowing easier coronary access (30, 31).

Regarding hard endpoints after TAVI in type 0 vs. type 1 BAV, the data are scarce and inconsistent. Jilaihawi et al. found that the patients with bicommissural non-raphe-type (i.e., type 0) BAV had higher procedural mortality than bicommissural raphe-type (i.e., “anatomical” type 1) BAV (9.5% vs. 0, *p* = 0.047), although no significant difference was detected at 30 days (13). Similarly, Yousef et al. showed that type 1 BAV with left and right cusp fusion was associated with markedly lower procedural, 30-day, and 1-year mortality, compared with other valve variants (*p* ≤ 0.05) (9). However, these mortality differences were driven by just several cases from the above early small-scale studies. Conversely, no significant differences in procedural or 30-day mortality were detected between the Sievers type 0 vs. type 1 BAV in the other five enrolled studies (8, 19–22). Notably, three of them reported no procedural death or 30-day death for the patients with Sievers type 0 BAV (20–22). Consistently, a similar mortality up to 5 years was demonstrated between the two BAV subsets in the patients receiving SAVR after adjusting for age, diabetes, and left ventricular ejection fraction (26). In line with these findings, we did not found marked differences in procedural death, 30-day all-cause death, or 30-day cardiac death between the two BAV morphologies in the pooled analysis.

In addition, our study has some limitations. The trials included were either small feasibility studies or large retrospective registries, with inconsistent inclusion and exclusion criteria, thus the selection bias was hardly avoidable. Most of the enrolled studies did not report calcification distribution on raphe or leaflet, or aortic annulus elliptic shape, unfavorable anatomies for TAVI in type 1, and 0 BAV (2, 32). The absence of these data precluded further subgroup analyses. Additionally, TAVI prosthesis might be constrained and under expanded in the patients with BAV with an asymmetrical aortic valvular complex, followed by accelerated deteriorating over time (24). However, bioprosthesis durability after TAVI in type 0 vs. type 1 BAV remained unknown due to the short-term follow-up.

## Conclusion

In the elderly severe AS population with low surgical risk, the patients with Sievers type 0 BAV seem to have higher mean aortic gradient and increased coronary obstruction risk, but otherwise similar procedural and 30-day outcomes after TAVI compared with type 1 BAV. However, the current patients with BAV that underwent TAVI were highly selected, and future studies should identify the BAV related optimal anatomies, refine sizing strategies, and best implantation techniques for TAVI.

## Data Availability Statement

The original contributions presented in the study are included in the article/supplementary material, further inquiries can be directed to the corresponding author/s.

## Author Contributions

YD, YZha, and YZho proposed the idea for the study and finished the study design. YY and SJ retrieved studies, collected and extracted data with disagreements resolved by YG and WH. YD, HS, and KH performed the meta-analysis and drafted the manuscript with a complete review by ZW and WL. All have authors read and approved the final manuscript.

## Funding

The National Key Research and Development Program of China (2017YFC0908800), the Beijing Municipal Administration of Hospitals' Mission plan (SML20180601), the Capital's Funds for Health Improvement and Research (FH 2020-2-2063) (KM200910025012), the Beijing Municipal Natural Science Foundation (7202041), and the Beijing Municipal Health Commission (jing19-15).

## Conflict of Interest

The authors declare that the research was conducted in the absence of any commercial or financial relationships that could be construed as a potential conflict of interest.

## Publisher's Note

All claims expressed in this article are solely those of the authors and do not necessarily represent those of their affiliated organizations, or those of the publisher, the editors and the reviewers. Any product that may be evaluated in this article, or claim that may be made by its manufacturer, is not guaranteed or endorsed by the publisher.
